# Cardiac Failure after Liver Transplantation Requiring a Biventricular Assist Device

**DOI:** 10.1155/2014/946961

**Published:** 2014-11-09

**Authors:** Rita Jermyn, Eiei Soe, David D'Alessandro, Julia Shin, William Jakobleff, Daniel Schwartz, Milan Kinkhabwala, Paul J. Gaglio

**Affiliations:** ^1^Division of Heart Failure, Department of Cardiology, Montefiore Medical Center, Albert Einstein College of Medicine, Bronx, NY, USA; ^2^Division of Hepatology, Department of Gastroenterology, Montefiore Medical Center, Albert Einstein College of Medicine, Bronx, NY, USA; ^3^Department of Cardiothoracic Surgery, Montefiore Medical Center, Albert Einstein College of Medicine, Bronx, NY, USA; ^4^Department of Pathology, Montefiore Medical Center, USA

## Abstract

Increased hepatic iron load in extrahepatic organs of cirrhotic patients with and without hereditary hemochromatosis portends a poorer long term prognosis after liver transplant. Hepatic as well as nonhepatic iron overload is associated with increased infectious and postoperative complications, including cardiac dysfunction. In this case report, we describe a cirrhotic patient with alpha 1 antitrypsin deficiency and nonhereditary hemochromatosis (non-HFE) that developed cardiogenic shock requiring mechanical circulatory support for twenty days after liver transplant. Upon further investigation, she was found to have significant iron deposition in both the liver and heart biopsies. Her heart regained complete and sustained recovery following ten days of mechanical biventricular support. This case highlights the importance of preoperatively recognizing extrahepatic iron deposition in patients referred for liver transplantation irrespective of etiology of liver disease as this may prevent postoperative complications.

## 1. Introduction

Patients with the heterozygous gene mutation for HFE or human hemochromatosis protein may demonstrate extrahepatic iron overload leading to organ impairment, particularly when associated with additional etiologies of chronic liver disease [1]. It has been observed that steatohepatitis and viral hepatitis may also contribute to abnormalities in control of iron homeostasis [1]. As an example, iron accumulation in extrahepatic sites such as the heart has been described in a series of patients with non-HFE associated iron overload [2]. This phenomenon has been associated with fatal cardiovascular complications after liver transplant [3]. Further exacerbating the complexity of extrahepatic iron overload is the potential for increased predisposition to infection with diminished cardiac output in the setting of stress or sepsis [1]. We report a patient with non-HFE hemochromatosis who developed profound heart failure after liver transplantation; this represents the first described case of cardiac iron deposition contributing to prolonged cardiogenic shock requiring mechanical circulatory support with eventual complete and sustained biventricular recovery.

## 2. Case Report

**Figure 1:**
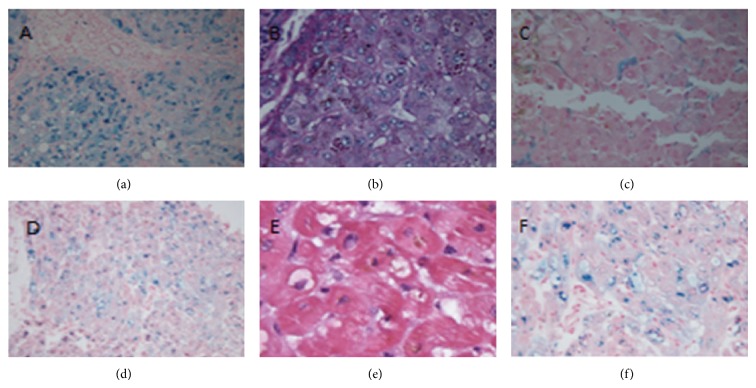
(a) Prussian-Blue stain showing iron granules in hepatocytes and bile duct epithelium in the explanted liver. (b) PAS-Diastase stain showing alpha 1 antitrypsin globules in periportal hepatocytes in the explanted liver. (c) Prussian-Blue stain showing iron granules in Kuppfer cells in the transplanted liver. (d) Prussian-Blue stain with extensive cytoplasmic iron in cardiac myocytes. (e) Hematoxylin and Eosin stain with intracytoplasmic hemosiderin in myocytes. (f) Prussian-Blue stain high power view of cytoplasmic iron in cardiac myocytes.

The patient is a 46-year-old Caucasian female with a history of non-HFE hemochromatosis, alpha 1 antitrypsin deficiency, and cavernous transformation of the portal vein, who presented to our institution for orthotopic liver transplantation. Pretransplant liver biopsy was not performed due to the patient's severe portal hypertension and risk of bleeding. Gene testing revealed she was compound heterozygous for the HFE gene (C282Y-H63D) with a low alpha 1 antitrypsin level, and Hepatitis B DNA and Hepatitis C RNA were negative. Serum iron was 104 mg/dL (normal range 55–155 mg/dL), transferrin was 136 mg/dL, ferritin was 1240 ng/mL, and percent iron saturation was 61% (normal range 15–50%). She underwent uncomplicated orthotopic liver transplantation; however 72 hours postoperatively she developed multidrug resistant* E. coli* and MRSA peritonitis. Her blood cultures remained negative. Liver explant showed 3+ iron predominantly within hepatocytes and occasional Kupffer cells with intracytoplasmic alpha-1 antitrypsin globules on periodic acid-Schiff staining, indicative of both iron overload and alpha 1 antitrypsin deficiency (Figure 1). Approximately 72 hours postoperatively, she developed hypotension required reintubation and vasopressor support. An echocardiogram revealed severe, nondilated biventricular failure with an ejection fraction of 10 percent. Prior cardiac workup included a dobutamine stress echocardiogram done six months prior to liver transplantation which showed no ischemia, normal ventricular anatomy, and a normal ejection fraction. The patient also had a transthoracic echocardiogram done two months preoperatively to assess portopulmonary hypertension after she was admitted for volume overload and dyspnea. This revealed a normal pulmonary artery pressure; however, her ejection fraction was moderately diminished globally to 45% percent. Additional cardiac assessment following transplantation revealed that the patient had a peak Troponin T of 0.15 ug/L, CPK 108 IU/L, and her EKG did not reveal any ST segment abnormalities or q waves. Serum ferritin was increased to 5800 ng/mL.

**Figure 2:**
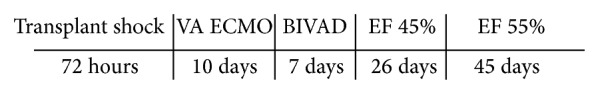
Timeline.

Following intubation and the initiation of vasopressor support, the patient continued to deteriorate with multiorgan failure, with continued depression of her cardiac function with an EF of ten percent. The patient was started on venoarterial ECMO support, and repeat echocardiogram revealed significant mitral regurgitation with poor LV unloading. After ten days of VA ECMO support, the patient did not appear to have any myocardial recovery on transthoracic echo. She was taken back to the operating room for temporary biventricular support. Cannulas were placed from right ventricular to pulmonary artery and left ventricle to the aorta. This device supports the right and left ventricles with a centrifugal pump that is magnetically levitated to reduce afterload of the failing left ventricle and provide preload from the failing right ventricle. The ECMO circuit was decannulated and the left ventricular apex was excised and sent for pathology. Pathology revealed abundant cytoplasmic iron demonstrated as hemosiderin. Biventricular support was continued for an additional seven days. On post-op day twenty-six her bedside transthoracic echocardiogram showed a dramatic improvement in left ventricular ejection fraction and EF of forty-five percent. The patient returned to the operating room for decannulation of her bivad where intraoperative TEE revealed only mildly reduced left ventricular and right ventricular ejection. After explantation of the bivad, the patient remained hemodynamically stable and a bedside transthoracic echocardiogram one week later revealed an ejection fraction of 55% with mild apical akinesis and normal right ventricular function. She is currently forty-five days post-op from orthotopic liver transplant and her cardiac functions remain normal (Figure 2).

## 3. Discussion

The cause of cardiogenic shock in this patient five days after orthotopic liver transplant appeared to be related to several contributing factors, potentially due to extrahepatic iron deposition and myocardial stunning from septic shock. This case demonstrates successful biventricular recovery after ECMO and bivad mechanical circulatory support. When analyzing the phenomenon of cardiogenic shock in this patient, the etiology and pattern were unclear. Typically, sepsis induced cardiomyopathy produces reversibility within seven to ten days and the typical pattern of remodeling on echocardiogram is that of anterior wall akinesis [4]. Our patient's cardiac recovery took significantly longer. In addition, we do not believe that the patient had significant intrinsic cardiac dysfunction preoperatively as pretransplantation dobutamine stress echocardiogram did not reveal any significant abnormalities, although on reanalysis her immediate preoperative echocardiogram began to display a slight decrease in left ventricular ejection fraction, potentially due to iron overload.

Iron overload has been classified as primary as in hereditary hemochromatosis or secondary due to chronic transfusion therapy and ineffective erythropoiesis. After identification of the human hemochromatosis protein (HFE) in 1996, it was noted that only a minority of patients were homozygous for the C282Y mutation, indicative of hereditary hemochromatosis [5]. However, it has been observed that patients with a heterozygous HFE gene mutation may also experience hepatic iron overload, particularly in the setting of other etiologies of liver disease. A number of theories have been proposed to explain enhanced iron deposition in this setting including increased absorption of iron because of ineffective erythropoiesis, hemolysis, and currently unrecognized mutations affecting iron metabolism. Intrahepatic portosystemic shunts have also been shown to result in hepatic iron overload [6]. Often alcoholic liver disease or chronic hepatitis C can be associated with hepatic iron overload. This is thought to be related to decreased hepcidin expression [7]. It has also been observed that individuals who display compound heterozygosity for the HFE gene mutation (C282Y/H63D) may develop hepatic as well as extrahepatic iron overload, the mechanism potentially related to additional gene mutations such as S65C mutation [8]. Any mutation in HFE causes increased transferrin which the intestines misinterpret as iron deficiency resulting in further increase in absorption of iron. The HFE gene also affects production of hepcidin in the liver which can further exacerbate inappropriate iron loading [8].

Iron overload may portend a poor prognosis following liver transplantation. Several studies have shown the 1- as well as 5-year survival after liver transplant is lower than expected in patients with severe iron overload when compared to patients without iron overload [9]. It has been hypothesized that iron deposition compromises the function of other organs such as the heart, pancreas, and brain. In addition, iron overload may predispose the patient to an increased risk of infection which is clearly problematic in an already immunocompromised host [9]. The pathophysiology of iron associated cardiac dysfunction has been well documented; by free radical mediated pathways, labile iron exceeds cellular antioxidant mechanisms and peroxidation of membrane lipids, cellular protein, and nucleic acids occurs [10]. Persistent free radicals affect the sarcoplasmic reticulum and mitochondria leading to calcium leakage into the cytoplasm [10]. Iron deposition in the heart first occurs in the ventricular followed by atrial myocardium. Increased ferrous iron transport through the L-type calcium channels of the heart results in impaired excitation-contraction coupling which may cause the systolic dysfunction appreciated in iron overload patients [9].

We hypothesize that our patient was already displaying subtle signs of cardiac dysfunction due to iron deposition preoperatively as she displayed mildly decreased left ventricular function on transthoracic echocardiogram. The most common cardiac manifestations associated with hereditary hemochromatosis are dilated cardiomyopathies and ventricular arrhythmias [9]. However, our patient was unusual in that, postoperatively, she displayed nondilated biventricular failure and was genotype negative for the homozygous HFE gene. Perhaps as a result of the already compromised myocardial function preoperatively due to iron, the patient was at greater risk of decompensation in the setting of sepsis induced myocardial stunning. This phenomenon of depressed myocardial function due to sepsis was first described by Maeder and Hunziker in 2009 [11]. A transiently decreased left ventricular ejection fraction with preserved stroke volume was often described in these patients with the average duration of abnormal function lasting four days and rebounding to normal by seven days. Patients with profound unremitting shock requiring mechanical circulatory support for twenty days exclusively due to sepsis are an extreme rarely reported condition [11]. Another potential factor in our patient to consider would be the physiologic circulatory state in some patients with portal hypertension, described as “cirrhotic cardiomyopathy” [12]. This phenomenon is most commonly but not exclusively seen in patients with alcoholic cirrhosis, characterized by elevated filling pressure, ventricular arrhythmias, and eventually systolic dysfunction. Diastolic dysfunction may be present in up to 25% of these patients after liver transplantation [12]. Given the frequency of preexisting cardiac disease and potential perioperative complications in cirrhotic patients, all patients considered for liver transplant undergo at minimum a noninvasive cardiac workup [12]. Our patient had no evidence of ischemia on dobutamine stress echo or elevated pulmonary pressure six months prior to transplant and thus cirrhotic cardiomyopathy was unlikely.

Therapy of cardiac iron overload has been previously described. For patients with acquired cardiac siderosis and systolic dysfunction, most frequently occurring in patients requiring chronic transfusions, there is literature showing there may be an improvement in ejection fraction with oral iron chelators. However, this was mostly reported in case reports and included both oral and intravenous chelation therapy in addition to typical heart failure medications for a minimum of 12 months [13].

This case was unusual for several reasons; it was remarkable that the patient was able to maintain a normal ejection fraction intra- and immediately postoperatively despite the dramatic circulatory changes that occur during and after liver transplantation. When the patient developed sepsis postoperatively, she developed profound biventricular failure, with eventual recovery using a combination of ECMO and biventricular mechanical support. This experience highlights the importance of recognizing hepatic iron deposition preoperatively as a risk factor for extrahepatic iron accumulation in end stage cirrhotic patients awaiting transplant. Iron deposition can enhance the risk of infection in immunocompromised patients and may lead to severe cardiac complications postoperatively. The role of preoperative chelation in patients with non-HFE associated iron overload needs to be explored.

## Abbreviations

Non-HFE: Nonhereditary hemochromatosis
HFE: Human hemochromatosis protein
VA ECMO: Venoarterial extracorporeal membrane oxygenation
BIVAD: Biventricular Assist Device.
